# Evolutionary Analysis of Novel Serine Proteases in the Venom Gland Transcriptome of *Bitis gabonica rhinoceros*

**DOI:** 10.1371/journal.pone.0021532

**Published:** 2011-06-24

**Authors:** Sakthivel Vaiyapuri, Simon C. Wagstaff, Robert A. Harrison, Jonathan M. Gibbins, E. Gail Hutchinson

**Affiliations:** 1 Institute for Cardiovascular and Metabolic Research, University of Reading, Reading, United Kingdom; 2 School of Biological Sciences, University of Reading, Reading, United Kingdom; 3 Alistair Reid Venom Research Unit, Liverpool School of Tropical Medicine, Liverpool, United Kingdom; 4 Blood Transfusion Research Group, King Saud University, Riyadh, Saudi Arabia; Instituto Butantan, Brazil

## Abstract

**Background:**

Serine proteases are major components of viper venom and target various stages of the blood coagulation system in victims and prey. A better understanding of the diversity of serine proteases and other enzymes present in snake venom will help to understand how the complexity of snake venom has evolved and will aid the development of novel therapeutics for treating snake bites.

**Methodology and Principal Findings:**

Four serine protease-encoding genes from the venom gland transcriptome of *Bitis gabonica rhinoceros* were amplified and sequenced. Mass spectrometry suggests the four enzymes corresponding to these genes are present in the venom of *B. g. rhinoceros*. Two of the enzymes, rhinocerases 2 and 3 have substitutions to two of the serine protease catalytic triad residues and are thus unlikely to be catalytically active, though they may have evolved other toxic functions. The other two enzymes, rhinocerases 4 and 5, have classical serine protease catalytic triad residues and thus are likely to be catalytically active, however they have glycine rather than the more typical aspartic acid at the base of the primary specificity pocket (position 189). Based on a detailed analysis of these sequences we suggest that alternative splicing together with individual amino acid mutations may have been involved in their evolution. Changes within amino acid segments which were previously proposed to undergo accelerated change in venom serine proteases have also been observed.

**Conclusions and Significance:**

Our study provides further insight into the diversity of serine protease isoforms present within snake venom and discusses their possible functions and how they may have evolved. These multiple serine protease isoforms with different substrate specificities may enhance the envenomation effects and help the snake to adapt to new habitats and diets. Our findings have potential for helping the future development of improved therapeutics for snake bites.

## Introduction

Snake venoms are complex mixtures of enzymatic and non enzymatic proteins, together with other components such as carbohydrates, lipids, nucleosides and metals. These function together to immobilize, kill and digest the prey [1] . Snake venoms have various envenomation effects and can be haemotoxic, myotoxic, neurotoxic and nephrotoxic towards prey and victims [2]. Snake venom serine proteases are a major component of venom and have been identified mainly in the venoms of snakes belonging to the viperidae family with a few occurring in members of the elapidae, colubridae and hydrophidae families [3]. Viper venom serine proteases (VVSPs) share similar nucleotide and amino acid sequences (with more than 60% sequence identity) and also three-dimensional structures, but have diverse functions. Generally they have haemotoxic effects, by affecting various stages of the blood coagulation system. They can act either as pro-coagulants via fibrin formation, factor V activation, kininogenolysis or platelet aggregation, or as anti-coagulants via fibrinolysis, plasminogen activation or protein C activation [4]. Several VVSP nucleotide sequences have been obtained by screening and sequencing venom gland cDNA libraries. Within these the 5′ untranslated regions (UTRs), N-terminal signal and activation peptide-coding sequences and 3′ UTRs have been found to be more highly conserved than the mature protein coding sequences [5]. Thus it is possible to identify novel VVSPs by screening cDNA libraries or amplifying a cDNA pool using specific primers for the conserved regions. Analysis of the nucleotide and amino acid sequences of these enzymes will help to understand their structure and function and provide insight into their evolution. A knowledge of the diversity of toxins and enzymes present within snake venom will also aid the development of novel treatments for snake bites. In this report, we describe the amplification and sequencing of four serine proteases from the venom gland transcriptome of the Gaboon viper *Bitis gabonica rhinoceros* using specific primers designed for the 5′ signal peptide coding sequence and the 3′ UTR and discuss the possible functions and evolution of these enzymes.

## Materials and Methods

### Materials used

Lyophilized venom of *B. g. rhinoceros* was obtained from the Liverpool School of Tropical Medicine, Liverpool, UK. The Illustra mRNA purification system was from GE Healthcare (Amersham, UK). Restriction enzymes, GoTaq® PCR Core System I and Wizard® SV Gel and PCR Clean-Up System were from Promega (Southampton, UK). The ZAP-cDNA synthesis kit was from Stratagene (La Jolla, USA), the TOPO TA Cloning® system and SimplyBlue™ SafeStain were from Invitrogen (Paisley, UK), and the QIAprep Spin Miniprep kit was from Qiagen (West Sussex, UK). TRI Reagent® and all other chemicals used were analytical grade from Sigma Aldrich (Poole, UK).

### Ethical statement

All activities conducted in the Alistair Reid Venom Research Unit at the Liverpool School of Tropical Medicine are licensed and approved by the UK Home Office (Project Licence 40/3216). Venom extraction from the snakes used in this study is no longer a procedure regulated by the Animals (Scientific Procedures) Act 1986. All efforts were made to minimize the suffering of animals.

### Venom gland cDNA synthesis

To enhance the expression of the venom gland genes, venom was extracted from a single specimen of *B. g. rhinoceros* maintained in the Liverpool School of Tropical Medicine three days before the dissection of venom glands. Total RNA was isolated from the venom gland tissues using TRI Reagent® and polyadenylated mRNA was purified using the Illustra mRNA purification system according to the manufacturer's protocols. cDNA was synthesized from the purified mRNA using the ZAP-cDNA synthesis kit.

### PCR amplification

Specific primers were designed for the 5′ signal peptide coding sequence and the 3′ UTR of the known *B. gabonica* serine protease I sequence (NCBI accession number: AAR24534) [6] and synthesized by Sigma Aldrich (Poole, UK). The sequences of the primers are: forward primer- 5′TGGTGTTGATCAGAGTGCT3′ and reverse primer- 5′ACAGAAGTACCAATAGAAGAGAAT3′. These primers were used to amplify the serine protease genes present in the venom gland cDNA by PCR (20 cycles) using denaturation at 94°C for 30 seconds, annealing at 52.7°C for 30 seconds, extension at 72°C for 1 minute and a final extension at 72°C for 10 minutes.

### Purification, cloning and sequencing of amplified DNA

The amplified product was analysed by 1% (w/v) agarose gel electrophoresis and the gel was sliced to purify the amplified DNA using the Wizard® SV Gel and PCR Clean-Up System. Eluted DNA was cloned into a TOPO TA Cloning® system according to the manufacturer's protocols. The recombinant colonies were selected and grown in LB broth and the plasmids were purified using a QIAprep Spin Miniprep kit. Restriction digest analysis was used to confirm the presence of inserts and the plasmids were sequenced using M13 forward and reverse primers (as these sites flank the multi cloning site of the TOPO vector) by Cogenics Limited (Essex, UK).

### Venom protein separation

The venom proteins were separated using a micro rotofor (Bio-Rad, Hemel Hempstead, UK) as described previously [7]. Briefly, 1 mg of *B. g. rhinoceros* venom was mixed with 3 ml of non-reducing rotofor buffer [7 M urea, 2 M thiourea, 10% (v/v) glycerol and 2.5% (v/v) amphoyltes (pI 3–10) in Milli-Q water] and loaded on to the focussing chamber. Isoelectric focussing was performed under cooling setting I (temperature between 4°C and 15°C) with the programmed electric field (150 V/2 W/20 mA for 15 minutes, 200 V/2 W/20 mA for 15 minutes, 300 V/2 W/20 mA for 20 minutes, 350 V/2 W/20 mA for 20 minutes and 400 V/2 W/20 mA for 60 minutes). 0.1 M orthophosphoric acid and 0.1 M sodium hydroxide were used as anode and cathode electrode buffers respectively. Twenty microlitres of separated rotofor fractions were used to analyse the serine protease activity and 10 µl were used for SDS-PAGE to analyse the protein separation patterns.

### SDS-PAGE and staining

Reducing SDS-PAGE was performed using standard techniques [8]. The gel was stained with SimplyBlue™ SafeStain and scanned using a Typhoon Trio variable mode imager (GE Healthcare, Amersham, UK) before excising the bands for mass spectrometry.

### Serine protease assay

Serine protease activity of rotofor separated factions was measured using a fluorescent substrate, Nα-benzoyl-L-arginine 7-amido-4-methylcoumarin.HCl (Arg-AMC) (B7260, Sigma Aldrich) as previously described [9]. Twenty microlitres of separated rotofor fractions were mixed with Arg-AMC (20 nM) along with trypsin and thrombin as positive controls and incubated at 37°C. The amount of 7-amido-4-methylcoumarin (AMC) released was measured at different time points using a spectrofluorimeter (FLUOstar OPTIMA, BMG Labtech, Offenburg, Germany) at an excitation wavelength of 366 nm and an emission wavelength of 460 nm. All measurements were performed in triplicate.

### In-gel digests, liquid chromatography-tandem mass spectrometry

In gel tryptic digestion was performed as described previously [10]. Tryptic digests were then reconstituted in 12 µl 0.1% TFA. Four microlitres of sample were loaded for 5 minutes at 30 µl/minute 0.2% TFA on a 10 mm trap column packed with 3.5 µm C_18_ particles (LC Packings/Dionex, Amsterdam, The Netherlands) and eluted in 0.2% formic acid for 10 minutes in a 2 to 15% CAN gradient followed by 80 minutes on a 15–40% gradient and finally 15 minutes on a 40–55% gradient at 250 nl/minute on a 15 cm×75 µm PepMap C_18_ reverse phase analytical column (3.5 µm particles; Packings/Dionex) using an Ultimate™ nLC system (LC Packings/Dionex). The LC system was coupled to a nESI-MS/MS 3D ion trap mass spectrometer (HCT Esquire; Bruker Daltonics, Bremen, Germany) and the nanoESI source was mounted with a 5 cm long stainless steel emitter (Proxeon). The LC system and the ion trap were controlled through HyStar™ and Esquire Control modules in the Compass™ software suite (Bruker Daltonics, Coventry, UK). Mass spectra were acquired from *m/z* 300 to 2,000 using parameters optimised at *m/z* 850 with the trap ion charge control set at 150,000 and a maximum acquisition time of 200 ms averaging three scans per spectrum. The three most abundant ions were selected for MS/MS, the isolation window was 4 *m/z* with a signal threshold of 5,000 and the fragmentation amplitude was 2 V. The selected precursor ions were actively excluded for 45s after two selections. Raw LC-MS/MS data were batch-processed in DataAnalysis 4.0 (Bruker Daltonics, Bremen, Germany). Up to 3,000 2^+^ and 3^+^ compounds (retention time restriction of 10–120 minutes) with a signal-to-noise ratio above 5 were extracted and exported as Mascot Generic Files (MGF).

For protein identification MGF files were submitted to Mascot2.2.2 (Matrix Science) on an in-house server. The Mascot search parameters were the following: 1.2Da error tolerance in MS mode and 0.4Da error tolerance in MS/MS mode, allowance of up to one tryptic missed cleavages and 2^+^,3^+^ and 4^+^ charged ions considered. Cysteine carbamidomethylation was set as a permanent modification and methionine oxidation was included as a variable modification.

### Sequence analysis

The nucleotide and translated protein sequences were analysed using DNASTAR Lasergene version 7 [11]. Multiple sequence alignment and pairwise alignments were performed using ClustalW [12] and EMBOSS [13] respectively. The sequence alignment figure was prepared using GeneDoc [14]. Glycosylation prediction was performed using NetNGlyc [15]. To generate the phylogenetic tree, sequences were aligned using ClustalW within MEGA 4 [16] using a gap opening penalty of 10 and a gap extension penalty of 0.1 for the initial pairwise alignment and gap opening penalty of 3 and gap extension penalty of 1.8 for the multiple alignment and the Gonnet protein weight matrix. The phylogenetic tree was generated within MEGA 4 using the neighbour-joining method and the Jones-Taylor-Thornton substitution model. The bootstrap test was done using 2000 replications. The sequence of bovine α-chymotrypsinogen (NCBI accession number: P00766) was used as an outgroup.

### Structural modelling of rhinocerases

Structural models of rhinocerases 2 to 5 were created using the IntFOLD server [17]. Good quality models were obtained for each sequence using the structure of rat trypsin (PDB code: 1co9) as a template. Models were visualised and compared with each other and the structures of bovine α-chymotrpysin (PDB code: 1YPH) and rat trypsin (PDB code: 1CO9) using PyMOL (DeLano Scientific).

## Results and Discussion

### Amplification of venom gland serine protease genes

In order to amplify the serine protease genes from the venom gland transcriptome of *B. g. rhinoceros*, specific primers were designed for the 5′ signal peptide coding sequence and 3′ UTR of the *B. gabonica* serine protease I sequence [6] as these regions are likely to be similar in other venom gland serine protease genes. The amplification resulted in DNA fragments with two different molecular masses, corresponding to around 900 bp and 1200 bp. This suggests the presence of venom gland serine protease genes which have similar 5′ signal peptide coding regions and 3′ UTRs but different lengths. Similar amplified products were obtained previously from the venom gland transcriptome of *Macrovipera lebetina* [5].

### Sequence analysis of rhinocerases 2 to 5

Sequencing of the amplified cDNA clones resulted in four distinct serine protease sequences, of lengths 906, 907, 1137 and 1179 bp. Each of these was confirmed by sequencing several clones. The 906 and 907 bp sequences were named rhinocerase 2 and rhinocerase 3, and the 1137 and 1179 bp sequences were named rhinocerase 4 and rhinocerase 5 respectively, following on from the naming of our previously purified serine protease, rhinocerase (now renamed rhinocerase 1) from the venom of *B. g. rhinoceros* [7]. (These have been deposited in the GenBank database under Accession Numbers FN868645 to FN868648.) Rhinocerases 2 and 3 are very similar to each other (nucleotide sequences 98% identical) and encode similar protein sequences (94% identical) with 259 amino acids (table 1). Similarly rhinocerases 4 and 5 are 90% identical to each other and encode proteins with 257 and 259 amino acids respectively which share 92% sequence identity. However the nucleotide and protein sequences of rhinocerases 2 and 3 are on average only 64% and 69% identical to those of rhinocerases 4 and 5.

**Table 1:** Features of the nucleotide and protein sequences of rhinocerases 2 to 5.

Sequence	Length of cDNA (bp)	Predicted coding region (bp)	Length of mature protein (aa)	Predicted mol. mass (kDa)	Predicted isoelectric point	No. of predicted N-glycosylation sites
Rhinocerase 2	907	3–779	236	26.28	8.1	1
Rhinocerase 3	906	3–779	236	26.27	8.1	2
Rhinocerase 4	1137	3–773	234	25.65	8.7	3
Rhinocerase 5	1179	3–779	236	26.05	8.9	2

The predicted coding regions, molecular masses and isoelectric points were obtained from DNASTAR Lasergene version 7. The potential N-glycosylation sites were predicted by NetNGlyc.

Rhinocerases 2 to 5 share several common features of viper venom serine proteases: 12 conserved cysteine residues and N-terminal signal (normally 18 amino acids) and activation peptides (normally 6 amino acids). Since the first nucleotide of the start codon was not included in our forward primer, our translated protein sequences show only 17 amino acids in the signal peptide region. However, the signal sequences of the native proteins would be expected to have 18 amino acids, similar to other VVSPs. As is common for VVSPs including rhinocerase 1 [7] and other venom enzymes [18], N-glycosylation sites were predicted in all four proteins (table 1) and thus the molecular masses of the native enzymes in the secreted venom may be higher than the predicted molecular masses. The predicted isoelectric points of rhinocerases 2 to 5 were between 8 and 9 which is clearly distinct from rhinocerase 1, the serine protease which we previously purified from the venom of this snake which had an isoelectric point of around 6, although the latter was measured for the glycosylated protein [7].

**Figure 1:**
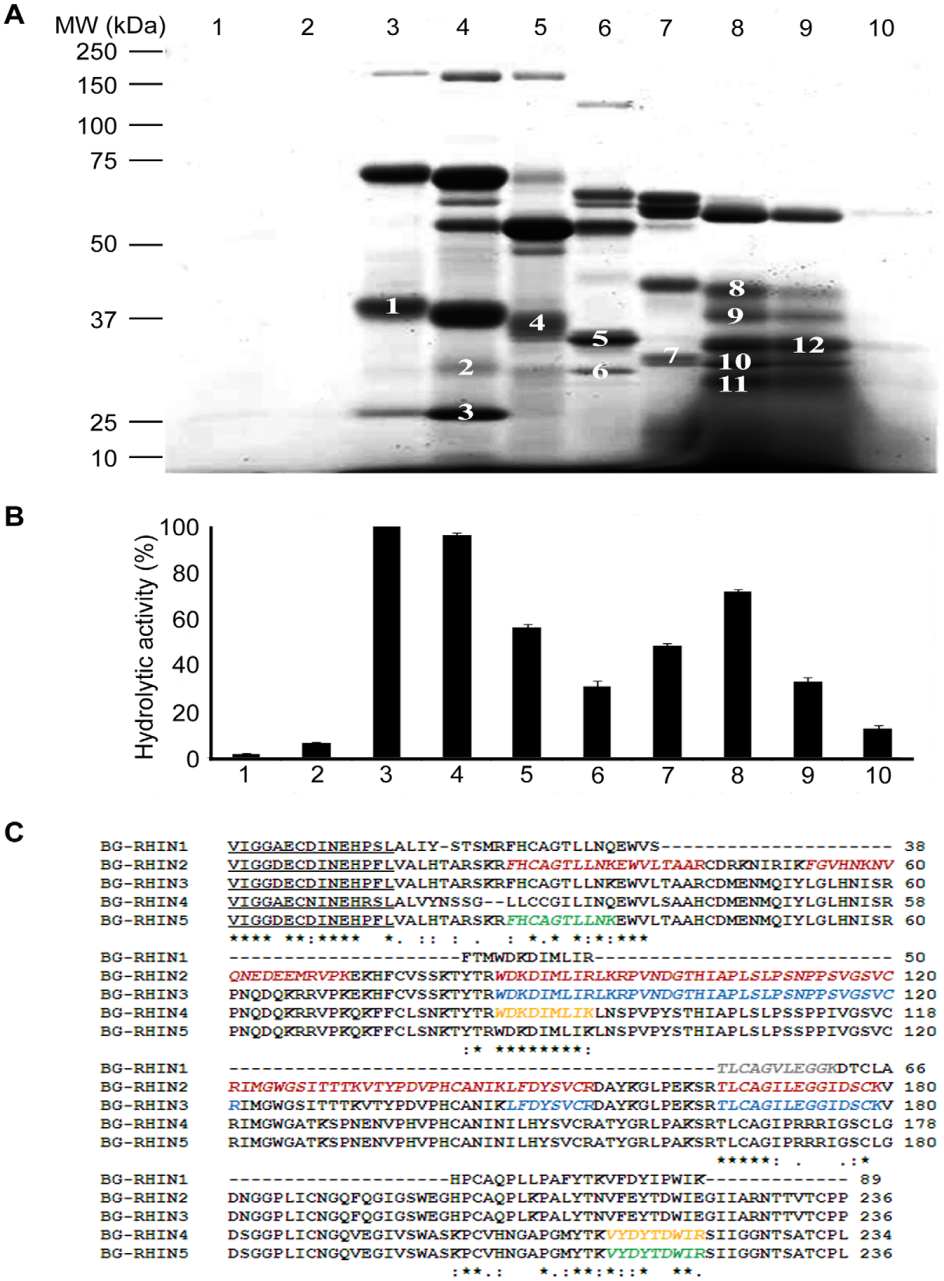
Identification of rhinocerases 1–5 in the venom of *B. g. rhinoceros*. *A.* 1 mg of venom was mixed with non-reducing rotofor buffer containing ampholytes with pI 3–10 and separated under non-denaturing conditions using a micro rotofor. In total 10 fractions (indicated by the numbers at the top of the gel) were collected. 10 µl of each fraction were run in SDS-PAGE (10%) and stained with SimplyBlue™SafeStain. The numbers mentioned on the gel bands represent the bands which were excised and used for mass spectrometry. *B*. 20 µl of each rotofor fraction were used to measure serine protease activity using Arg-AMC fluorescent substrate. The data represent the mean±S.D. (*n* = 3). The hydrolytic activity measured for fraction 3 was taken as 100%. *C*. The sequences of rhinocerases 2–5 were aligned with the partial sequence of rhinocerase 1 obtained previously. Gel bands 1, 7, 8, 11 and 12 (Fig. 1A) were analysed by mass spectrometry and the corresponding peptide sequences are shown in different colours (grey: band 1; red: band 7; yellow: band 8; blue: band 11; green: band 12) in italics on rhinocerase 1, 2, 4, 3 and 5 respectively. The N-terminal sequences of serine proteases in the venom of *B. g. rhinoceros* identified by proteomic analysis previously are underlined. The symbols ⋆, : and . indicate conserved residues, biochemically related residues and biochemically less related residues respectively.

Within a gel of rotofor-separated *B. g. rhinoceros* venom we have identified five distinct bands with molecular masses (figure 1A, bands 1, 7, 8, 11 and 12) and activities (figure 1B) consistent with serine proteases. The pIs of these proteins together with sequences derived from mass spectrometry analysis of tryptic digests of the gel bands are consistent with rhinocerases 1 to 5 (figure 1C). This suggests that, in addition to rhinocerase 1, rhinocerases 2 to 5 also exist in the venom of *B. g. rhinoceros*. This is also consistent with a proteomic analysis of *B. g. rhinoceros* venom which identified the N-terminal sequences of five distinct serine proteases [19]. One of these sequences is consistent with rhinocerases 2, 3 and 5, two (identical sequences) are consistent with rhinocerase 4, and a further sequence is consistent with our purified rhinocerase 1 [7] (underlined in figure 1C). The fifth sequence identified in the previous proteomic analysis must represent a serine protease distinct from any identified in our research so far. The only complete sequence of a serine protease within *B. gabonica* determined to date is that of serine protease I, which has been found at transcript level only [6]. The nucleotide sequence of this serine protease is almost identical to that of rhinocerase 2; there is one substitution in the mature protein-coding region at position 626 and a substitution in the 3′ UTR at position 895. The amino acid sequences of the corresponding proteins are identical, thus the nucleotide substitutions could represent synonymous mutations. Together these data are consistent with rhinocerases 2 to 5 representing novel venom serine proteases present in the venom of *B. g. rhinoceros*.

### Serine proteases in the *B. g. rhinoceros* transcriptome and their homologues

**Figure 2:**
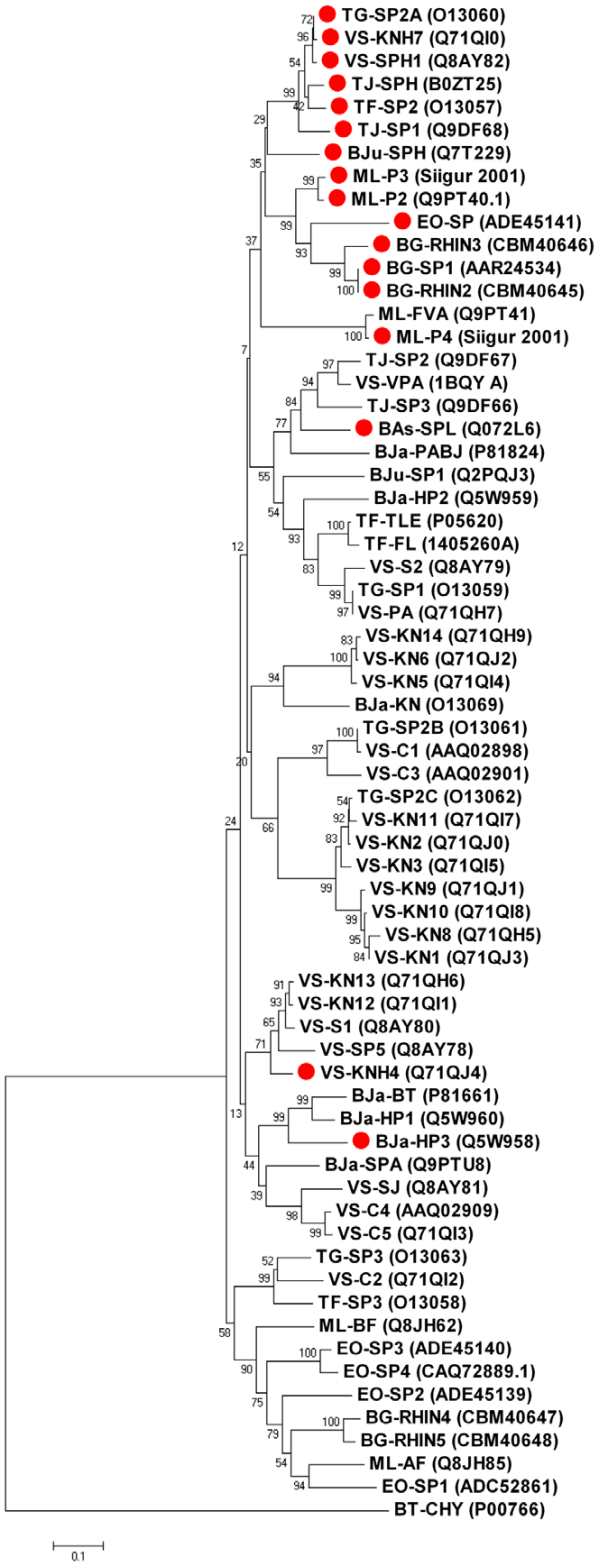
Phylogenetic tree showing relationship between serine protease homologues and serine proteases from the same snakes. 65 amino acid sequences from 10 snakes were included together with bovine α-chymotrypsinogen (NCBI accession number: P00766) which was used as an outgroup. The alignment was generated using ClustalW [12] within MEGA 4 [16] using a gap opening penalty of 10 and a gap extension penalty of 0.1 for the initial pairwise alignment, gap opening penalty of 3 and gap extension penalty of 1.8 for the multiple alignment and the Gonnet protein weight matrix. The phylogenetic tree was generated from this within MEGA 4 using the neighbour-joining method and the Jones-Taylor-Thornton substitution model. The bootstrap test was done using 2000 replications. In the diagram sequences are identified using a code which consists of up to 3 characters representing the snake name, (TG: *Trimeresurus gramineus*; VS: *Viridovipera stejnegeri*; TJ: *Trimeresurus jerdonii;* BJu: *Bothrops jararacussu* ; ML: *Macrovipera lebetina*; EO: *Echis ocellatus*; BG: *Bitis gabonica*; Bja: *Bothrops jararaca*; TF: *Trimeresurus flavoviridis;* BAs: *Bothrops asper*) followed by a dash and then up to 5 characters representing the protein name. Where possible NCBI accession numbers are also included. ML-P3 and ML-P4 sequences were obtained directly from the sequences named VLP3 and VLP4 in [5]. Red circles indicate the sequences with mutations to the catalytic triad.

Trypsin-like serine proteases share a catalytic triad which comprises His57, Asp102 and Ser195 (bovine α-chymotrypsinogen numbering). These residues are conserved in rhinocerases 4 and 5 and in the majority of known VVSPs. Thus rhinocerases 4 and 5 would be expected to be catalytically active. However rhinocerases 2 and 3 have His57Arg and Ser195Asn substitutions. Mutations to the catalytic triad residues have previously been observed in a small number of viper venom serine proteases [5], [6], [20], [21]. The majority of these sequences have been identified at transcript level only; prior to this study only one serine protease with catalytic triad substitutions has been identified within the venom of a snake [21]. Functional analysis of this protein did not detect any arginine esterolytic, fibrinolytic or proteolytic activity, and such sequences are commonly called serine protease homologues [21]. 15 complete amino acid sequences of viper venom serine proteases with mutations to the catalytic triad are available either in the literature or in the NCBI sequence database. These are derived from ten different snakes representing both Crotalinae and Viperinae sub-families of viper snakes. Phylogenetic analysis of the 65 VVSP sequences identified to date in these ten snakes shows that the majority of the serine protease homologues, including rhinocerases 2 and 3 cluster together, although one serine protease with a standard catalytic triad is also in this cluster (figure 2). The remaining three serine protease homologues occur individually within the phylogenetic tree but Wu *et al.*'s [21] earlier analysis concluded that only one sequence (KNH4 from *Viridovipera stejnegeri*) did not cluster with the main group of serine protease homologues. Our updated analysis suggests that the creation of serine proteases with mutated catalytic triads has occurred several times during the evolution of these sequences.

**Figure 3:**
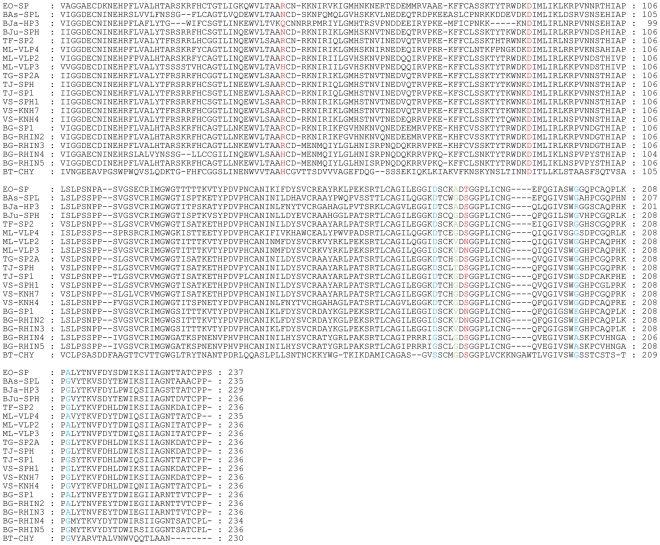
Amino acid sequence alignment of rhinocerases with other viper venom serine protease homologues. The alignment was created using ClustalW [12] and the figure was generated using GeneDoc [14]. The sequence of bovine α-chymotrypsinogen (NCBI accession number: P00766) (BT-CHY) was included to allow conventional serine protease residue numbering to be assigned. The catalytic triad residues are coloured red, the primary specificity pocket residues are coloured blue and residue 193, involved in the oxyanion hole is coloured green. BG-SP1: AAR24534; BG-RHIN2: CBM40645; BG-RHIN3: CBM40646; EO-SP: ADE45141; ML-P2: Q9PT40; TF-SP2: O13057; TJ-SPH: B0ZT25; TG-SP2A: O13060; VS-KNH7: Q71Q10; VS-SPH1: QAY82; TJ-SP1: Q9DF68; VS-KNH4: Q71QJ4; BJu-SPH: Q7T229; BJa-HP3: Q5W958; ML-P3 and ML-P4 from [5]; Bas-SPL: Q072L6; BG-RHIN4: CBM40647; BG-RHIN5: CBM40648.

**Figure 4:**
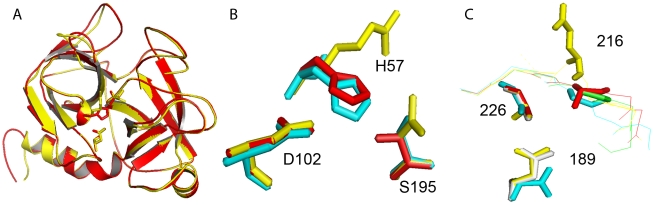
Structural models of rhinocerases. Structural models of rhinocerases 2 to 5 were created using the IntFOLD server [17] using the structure of rat trypsin (PDB code: 1co9) as a template. *A.* Schematic diagram showing the overall similarities in structure between rhinocerase 2 (yellow) and rhinocerase 4 (red). The side chain atom positions for the catalytic triad residues are included. *B*. Detailed view of the amino acids corresponding to the catalytic triad residues in rhinocerase 2 (yellow), rhinocerase 4 (red) and chymotrypsin (PDB code: 1yph; cyan). Rhinocerase 2 has substitutions for the serine and histidine residues. *C*. Detailed view of the main constituents of the S1 specificity pocket in rhinocerase 2 (yellow), rhinocerase 4 (red), chymotrypsin (cyan) and trypsin (pdb code: 1co9; green). In chymotrypsin these residues are: S189 at the base of the specificity pocket, with G216 and G226 at the sides. In trypsin D189 is at the base of the pocket, with G216 and G226. All images were generated using PyMOL.

Figure 3 shows an alignment of the sequences of the 15 serine protease homologues with all four rhinocerases and bovine α-chymotrypsinogen. The most frequently mutated residue within the catalytic triad is His57, which has been substituted by Arg in 13 sequences (including rhinocerases 2 and 3), by Asn in 2 sequences and by Gln in one sequence. Seven of the sequences have substitutions for the catalytic Ser195; in five proteins including rhinocerases 2 and 3 this has been substituted by Asn, one protein has a Ser195Pro mutation and one has a Ser195Thr mutation. In contrast, Asp102 is absolutely conserved in all the sequences. Structural models of the rhinocerases show their overall similarity, consistent with the sequence similarity of the enzymes (figure 4A), but differences in their active site regions. Asp102 is at almost identical positions in rhinocerases 2 and 4, and bovine chymotrypsin. The catalytic serine is at a very similar position in rhinocerase 4 and chymotrypsin, and the substituted Asn in rhinocerase 2 is also similarly located. However the substitution of the long arginine side chain instead of histidine at position 57 in rhinocerase 2 has resulted in a significant change to the orientation of the side chain (figure 4B).

Proteins with mutations to the catalytic triad are present in many enzyme families; indeed it has been estimated that up to 15% of the members of all encoded enzyme families may have lost their catalytic activity [22]. In many cases the inactive homologues are believed to have acquired alternative functions, such as competing with and antagonising the active proteases, or otherwise regulating their function. Within invertebrates, serine protease homologues have been shown to be involved in various defence responses [23]. However, it has been suggested that at least some invertebrate serine protease homologues are unlikely to bind peptide substrates by a canonical protease-like mechanism, though other potential protein binding sites have been suggested [24]. Within snake venom, catalytically inactive phospholipase A_2_ such as myotoxins II and IV from the venom of *Bothrops asper* are known to act as toxins and are thought to bind to their target membrane substrates in order to reduce their permeability control and cause subsequent necrosis [25]. Clearly, experiments to determine the function of the serine protease homologues within snake venom need to be performed, but it is possible that they could affect the physiology of victims or prey by binding irreversibly to substrates involved in blood coagulation and preventing their normal function.

There are also differences in the amino acids present in the primary specificity pockets of rhinocerases 2 to 5. The primary specificity pocket of trypsin-like serine proteases normally comprises Asp189, Gly216 and Gly226 (bovine α-chymotrypsinogen numbering) and these confer specificity towards basic residues at the P1 position of potential substrates. Rhinocerases 2 and 3 have Asp189, which might indicate specificity for basic residues but have Glu instead of Gly at position 216 and Ala instead of Gly at position 226. These substitutions are likely to restrict access to the specificity pocket, thus the binding specificity of rhinocerases 2 and 3 is not clear. Rhinocerases 4 and 5 are the only VVSPs represented in the sequence alignment which have substitutions at position 189: they have Gly at this position instead of the negatively charged Asp. This is an unusual substitution, which is shared by the human kallikrein KLK9 whose specificity is unknown [26]. The substitution might be expected to increase the size of the specificity pocket; *M. lebetina* α and β-fibrinogenases (ML-AF and ML-BF) also have Gly189 [27] and Siigur *et al.* [28] have reported that ML-AF hydrolysed the Tyr16–Leu17, Phe24–Phe25 and Phe25–Tyr26 bonds of the insulin B chain, suggesting that enzymes with Gly189 can cleave substrates with large hydrophobic residues at the P1 position. Unlike ML-AF rhinocerases 4 and 5 also have a Gly to Ala substitution at position 216 which may narrow the specificity pocket slightly. Again, this is similar to human KLK9 which has Gly216 but Ala226. Comparison of the positions and orientations of residues in the S1 specificity pockets of rhinocerases 2 and 4 with those of chymotrypsin and trypsin suggest that, the bottom of the specificity pocket is very similar in rhinocerase 2 and trypsin, while the glycine in rhinocerase 4 is uncharged and protrudes even less into the pocket. At position 226, the glycines and alanines in all four enzymes are very similarly located. In contrast, the substitution of the large negatively charged glutamic acid at position 216 clearly has a significant effect on the S1 pocket in rhinocerase 2 (figure 4C). However the primary specificity pocket is not the sole determinant of specificity. For serine proteases involved in coagulation, the importance of additional regions of the structure e.g. exosites in recognition of substrates is becoming increasingly recognised [29].Thus the precise specificity of these enzymes can only be determined experimentally. The potential role of exosites in binding to substrates also strengthens our suggestion above that the serine proteases homologues which lack the catalytic triad may still be capable of interfering with the coagulation cascade through exosite-mediated interactions.

A further interesting feature of these sequences is that Gly193, which is generally highly conserved in serine proteases and is involved in the oxyanion hole and in inhibitor binding, is substituted in 8 sequences: by Val in four sequences including rhinocerases 2 and 3, by Thr in one sequence and by Ala in three sequences. As was found for TSV-PA, a venom plasminogen activator from *Trimeresurus stejnegeri*, which has Phe at position 193 [30], bulky residues substituted for Gly193 may reduce the sensitivity of the proteins to inhibitors such as bovine pancreatic trypsin inhibitor as well as reduce their interaction with substrates.

### Evolution of rhinocerases 2 to 5

**Figure 5:**
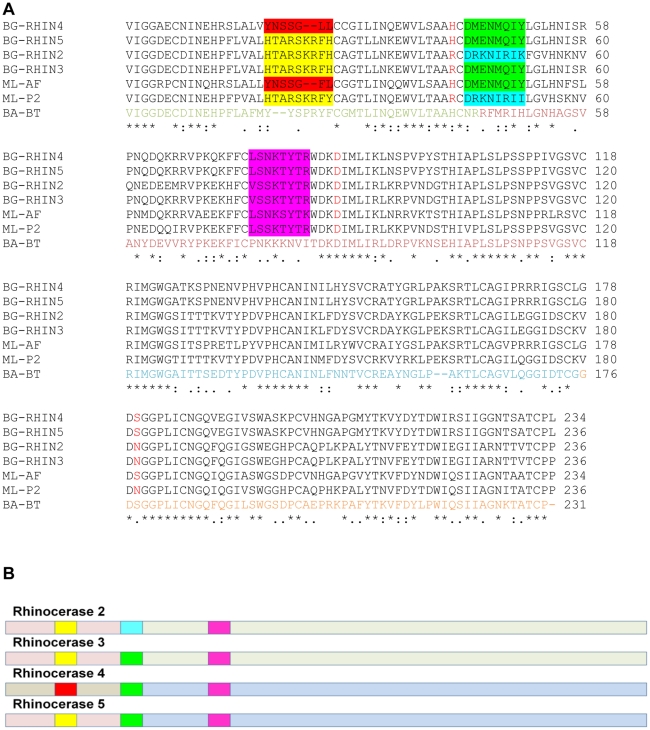
Evolution of rhinocerase enzymes. *A*. Amino acid sequence alignment of rhinocerases 2 to 5, *M. lebetina* α-fibrinogenase (ML-AF) and venom serine proteinase-like protein 2 (ML-P2), and *Bothrops atrox* batroxobin (BA-BT). The alignment was created using ClustalW. The amino acids in the batroxobin sequence are coloured according to the exons encoding them: amino acids encoded by exon 2 are coloured green, and residues encoded by exons 3 to 5 are coloured dark red, blue and orange respectively. The catalytic triad residues are coloured red in the *B.s gabonica* and *M. lebetina* sequences. Coloured shading is used to indicate the three surface segments within serine proteases identified by Doley et al. [26] as undergoing accelerated change (ASSET). Residues in the first surface segment are shaded yellow or red depending on sequence similarity; residues in the second segment are shaded green or turquoise and the residues in the third segment are shaded pink.*B*. Schematic diagram of rhinocerases 2 to 5 indicating the different regions described in the text. The light shading represents the regions of each sequence corresponding to exon 2 and exons 3 to 5. Rhinocerases 2, 3 and 5 have similar N-terminal regions corresponding to exon 2 (pink), while rhinocerase 4 has a different sequence in this region (grey). In the C-terminal regions, corresponding to exons 3 to 5, rhinocerases 2 and 3 are similar (pale green) and rhinocerase 4 and 5 are similar to each other (pale blue). The brighter shading represents the three surface segments as shown in *A*. The first surface segment is identical in rhinocerases 2, 3 and 5 (yellow), and different in rhinocerase 4 (red). The second segment is identical in rhinocerases 3, 4 and 5 (green) and different in rhinocerase 2 (turquoise), and the third segment is very similar in all 3 sequences (pink).

The four distinct serine protease genes which we have identified within the venom gland of *B. g. rhinoceros* provide further evidence for the existence of multiple isoforms of toxins within snake venom, yet the processes by which such isoforms have evolved are not yet fully understood. Accelerated evolution is well established as a mechanism for allowing changes at the individual amino acid level, and alternative splicing [5] and accelerated segment switch in exons to alter targeting (ASSET) [31], [32] have been recently proposed as additional mechanisms used for generating diversity in snake toxin sequences. A closer analysis of the sequences of rhinocerases 2 to 5 suggests that mechanisms such as alternative splicing and ASSET, together with individual amino acid mutations could have played a part in the generation of these isoforms (figure 5A,B).

Although in terms of overall similarity, rhinocerases 2 and 3 are very similar to each other and distinct from rhinocerases 4 and 5, sequence comparisons and BLAST searches of regions of the sequences corresponding to the exons as identified for the *Bothrops atrox* batroxobin gene (the only VVSP whose gene structure has been studied to date [33]) showed that rhinocerases 2, 3 and 5 have very similar N-terminal regions (up to residue 46 of rhinocerase 2; corresponding to exon 2 in batroxobin) which are most similar to the N-terminal region of *M. lebetina* venom serine proteinase-like protein 2 (NCBI accession number Q9PT40; indicated by ML-VLP2 in figures 3 and 5). Rhinocerase 4, on the other hand, has a distinctly different N-terminal region which is only 54% identical in sequence to the other rhinocerases and is most similar (81% sequence identity) to *M. lebetina* α-fibrinogenase (NCBI accession number Q8JH85, ML-AF).

A different pattern is seen within the C-terminal regions (residues 47 onwards of rhinocerase 2; corresponding to exons 3 to 5 in batroxobin). Two distinctly different versions of this region are also observed, however in part of the sequence rhinocerases 2 and 3 are very similar (92%) and rhinocerases 4 and 5 are identical to each other, but only around 60% identical to rhinocerases 2 and 3. The rhinocerase 2 and 3 sequences are most similar to *M. lebetina* venom serine proteinase-like protein 2 (ML-P2; 83%) while rhinocerase 4 and 5 are most similar to *M. lebetina* α-fibrinogenase (ML-AF) in this region (77%). Similar results were obtained when the C-terminal region was divided into separate regions corresponding to exons 3, 4 and 5 of batroxobin.

Thus rhinocerases 2 and 3 as a whole are similar to ML-P2, rhinocerase 4 as a whole is similar to ML-AF, while rhinocerase 5 has an N-terminal region very similar to rhinocerases 2 and 3 and a C-terminal region similar to rhinocerase 4. This could have been generated by splicing together exons corresponding to the N-terminus of rhinocerase 2 or 3 with exons corresponding to the C-terminal region of rhinocerase 4.

Doley *et al.* [32] identified three surface segments within serine proteases which seem to be undergoing accelerated change (ASSET). The first of these (residues 19–27) occurs within the N-terminal region corresponding to exon 2 in batroxobin and, consistent with the region as a whole, rhinocerases 2, 3 and 5 have identical sequences to each other within this segment, while the sequence of rhinocerase 4 is distinctly different. However it is interesting to note that His57 is also in the N-terminal region and this is conserved in rhinocerases 4 and 5 but mutated in rhinocerases 2 and 3. Thus this substitution must be an individual mutation occurring independently of any other mechanisms.

The second and third surface segments identified by Doley *et al.* [32] within serine proteases are located in the C-terminal region, corresponding to exons 3 to 5 in batroxobin. They found that the third surface segment was the most conserved, and indeed this segment is very similar (75% identical) in all four rhinocerase sequences, even though there are two distinctly different versions of the C-terminal region as a whole. The second surface segment (amino acids 45–52) is identical in rhinocerases 3, 4 and 5, with a different sequence in rhinocerase 2 (3 out of 8 residues matching). Thus this region could be switching independently of the C-terminal region as a whole, in which rhinocerases 2 and 3 are very similar.

Together these results suggest multiple mechanisms at work even in the evolution of this sub-set of *B. g. rhinoceros* serine proteases: mutations of individual amino acids clearly plays a role, but alternative splicing appears to be working alongside, and there are also changes within the surface segments identified by Doley *et al.* [32] as undergoing accelerated change within serine proteases. Further as yet unidentified mechanisms may also contribute to the generation of the diversity of toxins present in this and other snakes.

### Conclusions

In this study, we have reported the sequences of four serine proteases (rhinocerases 2 to 5) from the venom gland transcriptome of *B. g. rhinoceros*. These are clearly distinct from the rhinocerase 1 enzyme which we have recently purified from this venom. Mass spectrometry suggests the four enzymes corresponding to these genes are also present in the venom of *B. g. rhinoceros*. All four sequences share several common features of viper venom serine proteases: they have conserved signal and activation peptides, conserved cysteines and are predicted to be N-glycosylated. In addition these sequences have individual characteristics which are likely to affect their catalytic activity, substrate specificity and sensitivity to inhibitors. The variation within these sequences suggests that alternative splicing together with individual amino acid mutations may have been involved in their evolution. Changes within amino acid segments which were previously proposed to undergo accelerated change in venom serine proteases have also been observed. These multiple serine protease isoforms with different substrate specificities may enhance the envenomation effects and help the snake to adapt to new habitats and diets. A better understanding of the diversity of toxin isoforms present in individual snake venom will help in the design of improved therapeutics for treating snake bites.
